# Percutaneous Radiofrequency Ablation of Small (1–2 cm) Hepatocellular Carcinomas Inconspicuous on B-Mode Ultrasonographic Imaging: Usefulness of Combined Fusion Imaging with MRI and Contrast-Enhanced Ultrasonography

**DOI:** 10.1155/2018/7926923

**Published:** 2018-06-14

**Authors:** Min Woo Lee, Hyo Keun Lim, Hyunchul Rhim, Dong Ik Cha, Tae Wook Kang, Kyoung Doo Song, Ji Hye Min, Geum-Youn Gwak, Seonwoo Kim, David S. K. Lu

**Affiliations:** ^1^Department of Radiology and Center for Imaging Science, Samsung Medical Center, Sungkyunkwan University School of Medicine, Seoul 06351, Republic of Korea; ^2^Department of Health Sciences and Technology, SAIHST, Sungkyunkwan University, Seoul 06351, Republic of Korea; ^3^Department of Medicine, Samsung Medical Center, Sungkyunkwan University School of Medicine, Seoul 06351, Republic of Korea; ^4^Statistics and Data Center, Samsung Medical Center, Seoul 06351, Republic of Korea; ^5^Department of Radiological Sciences, David Geffen School of Medicine at UCLA, 757 Westwood Plaza, Los Angeles, CA 90095, USA

## Abstract

**Purpose:**

To assess usefulness of adding contrast-enhanced ultrasonography (CEUS) to fusion imaging (FI) for percutaneous radiofrequency ablation (RFA) of hepatocellular carcinomas (HCCs) inconspicuous on FI alone. Therapeutic outcomes of RFA under CEUS-added FI guidance for HCCs inconspicuous on FI alone were also evaluated.

**Methods:**

This prospective study was approved by the institutional review board and informed consent was obtained from all patients. Planning US was performed with FI for 126 patients with a single HCC (1–2 cm) to evaluate the feasibility of RFA by grading lesion conspicuity score using a four-point scale. RFA was performed under CEUS-added FI guidance for HCCs inconspicuous on FI alone. We evaluated how many HCCs initially inconspicuous on FI became conspicuous after adding CEUS. After CEUS-added FI-guided RFA, therapeutic outcomes including rates of technical success, primary technique efficacy, major complications, and local tumor progression were assessed.

**Results:**

After adding CEUS, 90.5% (19/21) of all tumors initially inconspicuous on FI became conspicuous, thus enabling direct targeting for RFA. Technical success and primary technique efficacy rates were 94.7% (18/19) and 100% (19/19), respectively. No major complications were observed after RFA. Cumulative local tumor progression rates after RFA were estimated to be 5.3%, 10.8%, and 10.8% at 1, 2, and 3 years, respectively.

**Conclusion:**

Adding CEUS to FI is useful for improving the conspicuity of HCCs inconspicuous on FI alone, thus enabling successful percutaneous RFA with excellent therapeutic outcomes.

## 1. Introduction

It can be challenging to ablate small hepatocellular carcinomas (HCCs) under B-mode ultrasonography (US) guidance as small HCCs sometimes have poor sonographic conspicuity [1]. Fusion imaging (FI) of B-mode US and pre-acquired computed tomography (CT)/magnetic resonance imaging (MRI) has emerged as a useful guidance modality for percutaneous radiofrequency ablation (RFA) of small HCCs [2–5]. FI increases the confidence of a subtle lesion by colocalizing it to pre-acquired CT/MRI. However, when a tumor is completely isoechoic compared to surrounding normal liver, FI can only estimate the location of what is in essence a “virtual target,” and incomplete ablation can occur even after FI-guided RFA [6]. This shortcoming is due to the inherent registration error that occurs when applying rigid registration to a deformable organ during motion.

Contrast-enhanced US (CEUS) using Sonazoid (gaseous perflubutane, GE Healthcare, New York City, NY) has emerged as a promising technique for localizing small HCCs and guiding percutaneous RFA [7, 8]. HCCs can be localized with high confidence using the post-vascular phase unique to Sonazoid [9].

CEUS is now often combined with FI for percutaneous RFA of challenging small HCCs [10, 11]. This combination has been shown to enhance the feasibility of percutaneous RFA for HCCs inconspicuous on FI alone [11, 12] and also better assess the ablation zone [13]. However, most of these studies had retrospective designs. They included heterogeneous mixtures of liver tumors and only evaluated the value of CEUS-added FI for localizing liver tumors without sufficient follow-up data [11, 12, 14, 15]. To our knowledge, there are no solid data regarding how many tumors initially inconspicuous on FI become conspicuous after adding CEUS, thus enabling direct targeting for RFA.

Therefore, the primary endpoint of this trial was to assess the value of adding CEUS to FI for improving lesion conspicuity and the technical feasibility of percutaneous RFA of HCCs (1–2 cm) inconspicuous on FI alone. The secondary endpoint was to assess the therapeutic outcomes of RFA under CEUS-added FI guidance for HCCs inconspicuous on FI alone.

## 2. Materials and Methods

### 2.1. Sample Size Estimation

The detection rate of very-early-stage HCC [Barcelona Clinic Liver Cancer (BCLC) stage 0, single nodule ≤ 2 cm] on FI has been reported to be approximately 85% [16]. Assuming that 15% are discordant pairs analyzed using the exact binomial test, a sample size of 101 is required with a 5% significance level and 80% power. Considering a drop-out rate of 20%, the minimum sample size was determined to be 126.

### 2.2. Patients

This prospective study was approved by the Institutional Review Board of Samsung Medical Center, a tertiary referral center. Informed consent was obtained from all patients prior to enrollment. Between August 2013 and January 2015, a total of 126 patients satisfying the following inclusion criteria underwent planning US to assess the feasibility of percutaneous RFA in our department. Inclusion criteria were as follows: (a) a treatment-naïve, single HCC 1–2 cm in diameter on contrast-enhanced MRI with gadoxetic acid (Gd-EOB-DTPA), (b) classification as Child-Pugh class A or B, (c) normal coagulation status, and (d) absence of vascular invasion and extrahepatic metastasis on MRI at the time of diagnosis. Exclusion criteria were as follows: (a) patients who did not undergo CEUS despite inconspicuous tumors on FI, (b) artificial ascites used instead of CEUS to improve sonographic window, (c) RFA infeasible due to reasons other than lesion conspicuity, (d) other treatments performed instead of RFA, and (e) patients who did not agree to participate in this study or who were lost to follow-up.

Treatment modality was determined based on recommendations from a multidisciplinary tumor board. Among these 126 patients, 34 patients were determined to have HCCs inconspicuous on FI alone; 19 of these patients who finally underwent percutaneous RFA under CEUS-added FI guidance were included in the evaluation of therapeutic outcomes after RFA.

Diagnosis of HCC was based on typical imaging features according to the American Association for the Study of Liver Disease guidelines [20] obtained on MRI. Scans were acquired using a 3.0-T system (Intera Achieva 3.0-T, Philips Healthcare, Best, The Netherlands) equipped with a 32-channel phased-array coil.

### 2.3. Planning US with Fusion Imaging

**Figure 1:**
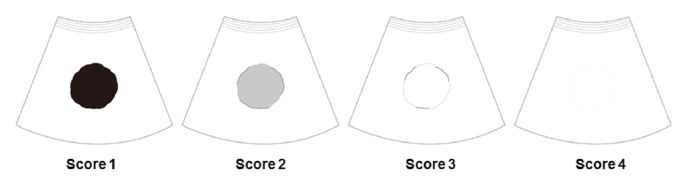
A schema showing the conspicuity score.

Planning US was performed to evaluate whether percutaneous RFA was feasible by one of three experienced radiologists (H.K.L., H.R., and M.W.L. with 15, 15, and 10 years of clinical experience with percutaneous RFA, respectively). For planning US, FI (Volume Navigation; GE Healthcare, Waukesha, WI) of the LOGIQ E9 system (GE Healthcare) was routinely used with a 1–5-MHz convex probe regardless of the initial US conspicuity of the tumor. The process of image fusion between B-mode US and MRI was similar to the methods of previous studies [2, 16]. After image fusion, the HCC conspicuity score on B-mode US was graded by the radiologist performing planning US using a four-point scale at the time of US examination (Figure 1 and Table 1). Lesions with a score of 1 or 2 were regarded as conspicuous HCCs, while those with a score of 3 or 4 were regarded as inconspicuous HCCs. The technical feasibility of RFA was also evaluated based on the expected electrode path, adjacent organ vulnerable to thermal injury, and heat-sink effect [17].

### 2.4. Percutaneous RFA

Percutaneous RFA was performed using a 200-W RF generator (VIVA RF system; STARmed, Goyang, Korea) and a length-adjustable RF electrode (Proteus; STARmed) by the radiologists who performed planning US. Additional CEUS was applied for HCCs inconspicuous on FI to increase lesion conspicuity, and RFA was performed if feasible under CEUS-added FI guidance. When CEUS was added to FI, image fusion between B-mode US and MRI was first performed to estimate the location of the index tumor before applying CEUS. Then, the US image was switched from B-mode to CEUS mode and displayed side by side with the fused MRI. Contrast harmonic imaging technique with a default setting of a mechanical index level of 0.24 was used. The focal zone was placed in the posterior margin of the liver. Sonazoid was injected intravenously at a dose of 0.015 mL/kg, followed by a flush with 10 mL of normal saline. Images were obtained during each phase: arterial (10–40 seconds after contrast injection), portal (60–90 seconds), and delayed phase (3 minutes) and post-vascular phase (more than 10 minutes) [11]. If the index tumor was not clearly identified on the arterial phase but was visible on the post-vascular phase, Sonazoid was reinjected in the post-vascular phase to visualize arterial enhancement of the target lesion [9]. The conspicuity score of the index tumor after adding CEUS was reassessed by the aforementioned scoring system based on the post-vascular phase as the temporal window of the post-vascular phase is long and the RF electrode is inserted during the phase. When the index tumor was definitely unidentifiable even after adding CEUS, percutaneous RFA was not attempted. Instead, an alternative treatment or imaging follow-up was considered.

The technical goals of RFA were to eradicate the tumor and to achieve a minimum 0.5-cm ablative margin where feasible. The energy deposition algorithm was based on the manufacturer's recommended protocol. Overlapping ablation was performed when needed. Although the standard ablation time recommended by the manufacturer was 12 minutes, RFA was finished earlier when the echogenic zone created was large enough to achieve a sufficient ablative margin or collateral thermal injury was suspected. The electrode path was cauterized during electrode removal at the end of the ablation session to avoid tract bleeding or tumor seeding.

### 2.5. Assessment of the Effects of Adding CEUS to FI and the Therapeutic Efficacy of RFA

For tumors inconspicuous on FI alone, we evaluated how initially inconspicuous HCCs became conspicuous after adding CEUS to FI, thus enabling direct targeting of percutaneous RFA. The rate of conspicuous HCCs on FI was compared with that on CEUS-added FI. Lesions conspicuous on FI alone were also regarded as conspicuous on CEUS-added FI, even though additional CEUS was not performed.

For patients who underwent RFA, therapeutic efficacy and complications were assessed using multiphase liver CT obtained within 12 hours after RFA. In addition, chest radiography, multiphase liver CT or MRI, and laboratory tests including serum *α*-fetoprotein were performed one month after initial discharge and every 3–4 months thereafter. Assessments were performed according to standardized terminology and reporting criteria for image-guided tumor ablation [21]. Technical success was defined as when the index tumor was treated according to our treatment protocol and was covered by ablation zone completely on the immediate post-RFA CT images [21]. The treatment course included all RFA sessions performed within one month to eradicate any residual tumor. Primary technique efficacy was assessed based on follow-up CT images taken one month after ablation. Local tumor progression (LTP) was defined as when foci of untreated disease appeared in tumors that were initially considered to be completely ablated on follow-up CT or MRI. A major complication was defined as any event that resulted in substantial morbidity and disability, an increased level of care, or substantial lengthening of hospital stay [21]. All other complications were regarded as minor.

### 2.6. Statistical Analysis

The conspicuity scores of inconspicuous HCCs on FI alone before versus after adding CEUS were compared using the Wilcoxon signed rank test. The rate of conspicuous HCCs on FI alone was compared with the rate of conspicuous HCCs on CEUS-added FI. Improvement of conspicuity score with the addition of CEUS was tested with the exact binomial test. Cumulative LTP rates of CEUS-added FI-guided RFA were estimated using the Kaplan-Meier method. All statistical analyses were performed using the SPSS Statistics software package (version 18.0; SPSS, Chicago, IL). A* p* value less than 0.05 was considered statistically significant.

## 3. Results

### 3.1. Patient and Lesion Characteristics

**Figure 2:**
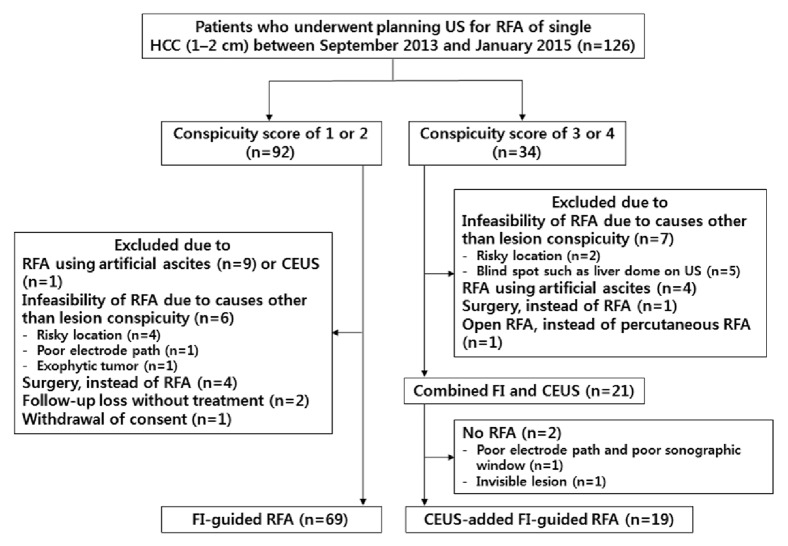
Flow diagram of the patient enrollment process. US = ultrasonography; HCC = hepatocellular carcinoma; RFA = radiofrequency ablation; FI = fusion imaging; CEUS = contrast-enhanced ultrasonography.

**Figure 3:**
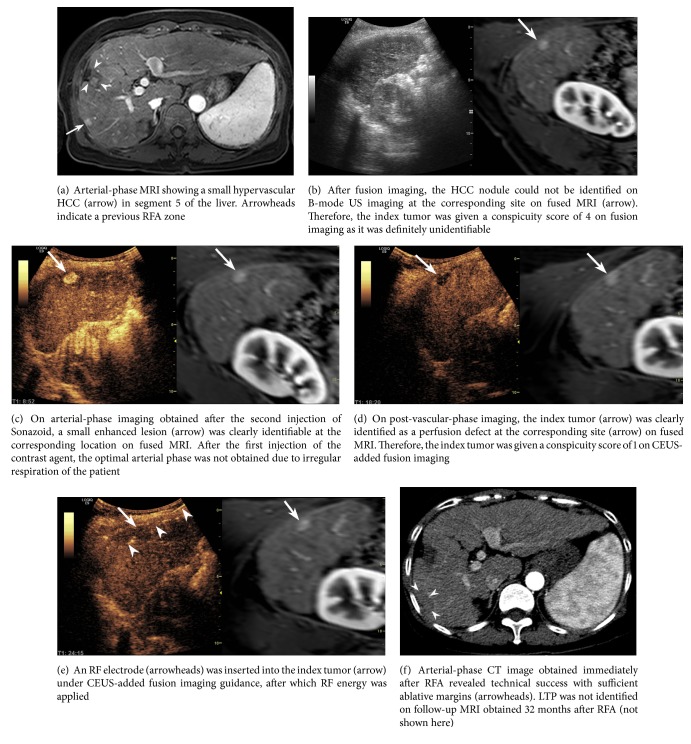
Images from a 59-year-old woman with a 1.1-cm HCC and hepatitis B virus-related liver cirrhosis who had previously undergone percutaneous RFA of HCC.

Of 126 patients, 92 (73.0%) patients had a lesion conspicuity score of 1 or 2, whereas 34 (27.0%) patients had a conspicuity score of 3 or 4 on FI (Figure 2). Of the 34 patients with a score of 3 or 4, 13 did not undergo CEUS due to the following reasons: percutaneous RFA was infeasible due to reasons other than lesion conspicuity (*n* = 7); artificial ascites fluid was used to enhance lesion conspicuity (*n* = 4); or other treatments were performed instead of percutaneous RFA (*n* = 2). The remaining 21 patients with conspicuity score of 3 or 4 also underwent CEUS (Figure 2). The conspicuity score of the 21 HCCs (mean ± standard deviation [SD], 1.2 ± 0.2 cm; median, 1.1 cm; range, 1.0–1.8 cm) was significantly higher after adding CEUS (before CEUS; median, 3; range, 3–4 versus after CEUS; median, 1; range, 1–4;* p* < 0.001) (Figure 3). Of the 21 lesions inconspicuous on FI alone, 19 (90.5%) became conspicuous after adding CEUS (16 on both arterial and post-vascular phase, two in post-vascular phase, and one in arterial phase) (Table 2). The rate of conspicuous HCCs on CEUS-added FI was significantly higher than that on FI alone [(92+19)/(92+21), 98.2% versus 92/(92+21), 81.4%;* p *< 0.001].

Among the 21 patients who underwent CEUS-added FI, one underwent chemoembolization instead of RFA due to poor electrode path and poor sonographic window due to perihepatic fat thickness, even though the index tumor was identified after adding CEUS (conspicuity score: 2). Another patient did not undergo RFA due to invisibility of the target lesion, even after adding CEUS. The latter patient was followed up with CT/MRI and the lesion had disappeared on 8-month follow-up MRI, indicating that it had been a pseudolesion. Eventually, direct targeting for percutaneous RFA was possible in 90.5% (19/21) of tumors initially inconspicuous on FI alone after adding CEUS (Figure 2).

### 3.2. Percutaneous RFA and Its Therapeutic Outcomes

The baseline characteristics of the 19 patients who underwent RFA under CEUS-added FI guidance are summarized in Table 3. Tumor size was 1.2 ± 0.2 cm. After RFA, a residual tumor was detected in one patient at CT, requiring a second ablation session to complete the treatment course. In this patient, the tumor (1.2 cm in diameter) was located in the subcapsular area of segment 5, just below the right rib. Although the conspicuity score of the tumor increased from 4 to 1 after adding CEUS, the sonographic window was poor due to the overlying rib shadow.

**Figure 4:**
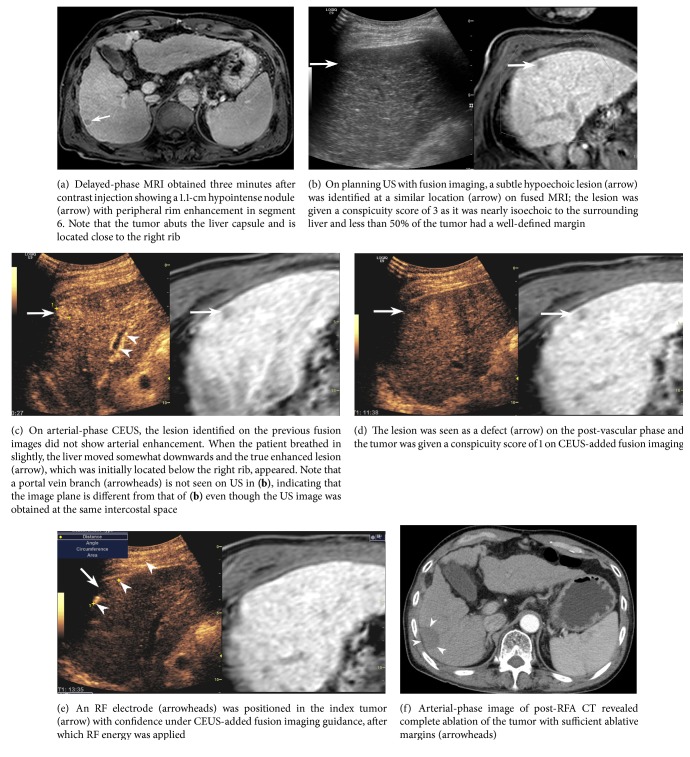
Images from a 65-year-old man with a recurrent HCC and hepatitis B virus-related liver cirrhosis who had previously undergone chemoembolization for HCC.

Meanwhile, incomplete ablation was avoided by adding CEUS in one case where a subtle lesion on FI was initially thought to be a true lesion that corresponded to the tumor nodule on MRI (Figure 4).

The therapeutic outcomes are summarized in Table 4. The technical success rate was 94.7% (18/19). No major complications were identified after CEUS-added FI-guided RFA. No residual tumors were identified in any patient on CT obtained one month after RFA (primary technique efficacy rate: 100%, 19/19). The cumulative LTP rates after CEUS-added FI-guided RFA were estimated to be 5.3%, 10.8%, and 10.8% at 1, 2, and 3 years, respectively.

## 4. Discussion

In general, FI can enhance the detectability of small HCCs inconspicuous on B-mode US and reduce false-positive detection of HCCs on B-mode US [16, 22]. In a previous study [16], false-positive detection on B-mode US was as high as 9.1% (9 of 99) for HCCs (1–2 cm) based on FI findings. In this context, we have adopted FI as the routine guiding modality for percutaneous RFA of HCCs. However, FI is not always sufficient for localizing small HCCs. Actually, 31.3% (76/243) of HCCs (mean size, 1.5 cm; range, 0.3–5.0 cm) were invisible on FI in a recent study [5].

Our study demonstrated that adding CEUS to FI improved the conspicuity of HCCs inconspicuous on FI of real-time US and MRI (Table 2). Consequently, it enabled percutaneous RFA of HCCs inconspicuous on FI alone in most cases (90.5%, 19/21). Moreover, unnecessary RFA was avoided in one case, which was later identified as a pseudolesion based on added CEUS and follow-up imaging findings. Furthermore, therapeutic outcomes including rates of technical success, primary technique efficacy, major complications, and LTP were excellent. Therefore, we propose that adding CEUS is a valuable strategy for accurate guidance of RFA for small (1–2 cm) HCCs inconspicuous on FI alone.

FI has been used in combination with CEUS for guidance of percutaneous RFA of HCCs in previous studies [10–12, 15, 23]. However, these studies were limited by selection bias due to their retrospective nature. Moreover, although they demonstrated the technical feasibility of combined FI and CEUS guidance, few studies have evaluated long-term therapeutic outcomes such as LTP rate [10]. When applying both FI and CEUS, no standardized method has been suggested by any society of interventional radiology or international working group on image-guided tumor ablation. In one study, CEUS was applied first, followed by FI [14], whereas in other studies, FI was applied first, followed by CEUS [11, 15, 23]. Both methods have also been used in a single study [10]. Although no data have been obtained to evaluate which method is better, we propose that FI be applied first because it requires no additional cost related to contrast agents in most cases. As demonstrated in our study, many small HCCs can still be identified using only FI. In addition, the overall procedure time of CEUS-guided RFA can be substantially decreased if FI is applied first. This is because the location of the target lesion can be estimated based on fused CT/MRI, which obviates the need for a second injection of Sonazoid to obtain the optimal arterial phase of the target lesion [11]. Moreover, when FI is applied first, image fusion can be performed within 3–4 minutes using manual registration methods [2, 5]. Nowadays, automatic registration methods facilitate easier and faster image fusion [24, 25]. Therefore, this procedure is much faster than typical CEUS performed using Sonazoid, in which a waiting period of about 10 minutes is needed after the initiation of contrast agent injection to obtain optimal post-vascular phase imaging.

Tumor size is one of the most important factors affecting lesion conspicuity on both B-mode US and FI of B-mode US and CT/MRI [1, 16, 26]. As expected, the mean size of tumors inconspicuous on FI was only 1.2 cm in our cohort. However, the rate of primary technique efficacy after percutaneous CEUS-added FI-guided RFA was 100% (19/19) and the LTP rate at 3 years was 10.8%. This implies that small HCCs (BCLC stage 0) can be ablated effectively once they are accurately localized.

According to a previous study [6], subphrenic or subcapsular tumor location is an important factor affecting mistargeting after FI-guided RFA of HCCs. Peripheral tumor location implies that large anatomic landmarks such as the portal vein branch cannot be used to locate the tumor. In addition, some tumors can be obscured by the rib shadow. Moreover, liver deformation and movement due to a patient's breathing or heart beat may be more apparent in peripheral liver than in central liver [6, 27]. This technical difficulty of percutaneous RFA of tumors in peripheral locations was also observed in our study. Residual tumor was noted in one patient with a tumor located in the subcapsular area, just below the right rib. Nevertheless, mistargeting or incomplete ablation can be avoided after CEUS, as seen in one of our cases (Figure 4).

Our study did have several limitations. First, this was a single-center study conducted at a tertiary academic hospital with a large volume of RFAs. In general, outcomes after RFA depend on operator experience. Also, 75% (66/88) of the study population had hepatitis B-related liver disease; thus, the results of this study may not be generalizable to other institutions or countries where hepatitis B virus is not the primary cause of liver cirrhosis or HCC. Second, lesion conspicuity was graded by one of three experienced radiologists and interobserver agreement was not assessed because the conspicuity score was graded in a prospective manner at the time of US examination. Third, more patients were excluded than expected before initiating the study. For example, four patients in whom artificial ascites was used during RFA were excluded from our final patient population because artificial ascites can enhance lesion conspicuity by improving the sonographic window [28]. Since we sought to determine the direct effect of CEUS on lesion conspicuity during percutaneous FI-guided RFA, this exclusion criterion may not have significantly affected our study outcome. Fourth, CEUS was selectively applied to a small number of patients with HCCs inconspicuous on FI alone because FI was sufficient for guidance of RFA of HCCs in many cases [5, 16]. Therefore, comparing the rate of conspicuous HCCs between FI alone and CEUS-added FI may yield limited insight. Applying CEUS to the entire study sample could have provided a more comprehensive understanding of the advantages and disadvantages of combined FI and CEUS.

In conclusion, adding CEUS to FI significantly improved the conspicuity of HCCs inconspicuous on FI alone, which enabled direct targeting for percutaneous RFA. This led to successful percutaneous RFA with excellent therapeutic outcomes.

## Figures and Tables

**Table 1:** Scoring criteria for evaluation of tumor conspicuity.

Score	Criteria
1	The echogenicity of the index tumor is definitely different from that of the surrounding liver and more than 90% of the tumor has a well-defined margin
2	The echogenicity of the index tumor is slightly different from that of the surrounding liver and more than 50% of the tumor has a well-defined margin
3	The index tumor is nearly isoechoic to the surrounding liver and less than 50% of the tumor has a well-defined margin
4	Definitely unidentifiable

This scoring system was described in our previous study [11] and was established after reviewing previous relevant studies regarding planning US for RFA and making necessary updates [17–19].

**Table 2:** Conspicuity score before and after CEUS in the 21 patients with fusion imaging-inconspicuous HCCs.

Before CEUS	After CEUS
3 (*n* = 13)	1 (*n* = 8)
	2 (*n* = 4)
	3 (*n* = 1)

4 (*n* = 8)	1 (*n* = 4) ^a^
	2 (*n* = 3) ^b^
	4 (*n* = 1) ^c^

CEUS = contrast-enhanced ultrasonography; HCC = hepatocellular carcinoma.

^a^ Residual tumor was noted after the first RFA session in one patient in whom the tumor was located in the subcapsular area in segment 5, just below the right rib. Technique efficacy was achieved after the second RFA session.

^b^ In one patient, although the lesion was identified after CEUS was added, both the expected electrode path and sonographic window were poor. Therefore, chemoembolization was performed instead of percutaneous RFA.

^c^ In this patient, the target lesion was not identified on the arterial or post-vascular phase. Therefore, instead of treatment, this patient was followed up with CT/MRI. Eventually, the lesion disappeared on 8-month follow-up MRI, indicating that it was a pseudolesion.

**Table 3:** Baseline characteristics of 19 patients who underwent percutaneous CEUS-added FI-guided RFA.

Characteristics	*n* = 19
Age (mean ± SD, y) (range)	63.9 ± 9.3 (50–81)
Sex (female) [number (%)]	5 (26.3)
Etiology (HBV/HCV/other) [number (%)]	10 (52.6)/2 (10.5)/7 (36.8)
Liver cirrhosis [number (%)]	10 (52.6)
HCC history [number (%)]	
None	3(15.8)
Resection	3(15.8)
RFA	2(10.5)
TACE	6(31.6)
Other	5(26.3)
Child-Pugh class (A/B) [number (%)]	19 (100)/0 (0)
Albumin (mean ± SD, g/dl)	4.1 ± 0.4
Total bilirubin (mean ± SD, mg/dl)	1.0 ± 0.5
PT (mean ± SD, INR)	1.14 ± 0.11
Serum AFP (mean ± SD, ng/ml)	8.5 ± 7.3
Tumor size (mean ± SD, cm) (range)	1.2 ± 0.2 (1.0–1.8)
Segment [number (%)]	
I	0 (0)
II	1 (5.3)
III	2 (10.5)
IV	1 (5.3)
V	6 (31.6)
VI	3 (15.8)
VII	4 (21.1)
VIII	2 (10.5)
Subcapsular location^a^ (yes) [numbers (%)]	5 (26.3)
Subphrenic location^b^ (yes) [numbers (%)]	5 (26.3)
Time interval between MR imaging and planning US (mean ± SD, days) (range)	12.4 ± 6.8 (2–31)
Time interval between planning US and RFA (mean ± SD, days) (range)	12.9 ± 8.0 (0–32)
Conspicuity score (median, range)	3, 3–4 (before CEUS)
	1, 1–4 (after CEUS)
Follow-up after RFA (median, range) (months)	30.2, 14.0–36.9

Data are presented as the number of patients or tumors with percentages in parentheses, unless otherwise specified.

AFP = *α*-fetoprotein; FI = fusion imaging; HBV = hepatitis B virus; HCV = hepatitis C virus; INR = international normalized ratio; PT = prothrombin time; RFA = radiofrequency ablation; TACE = transcatheter arterial chemoembolization; SD = standard deviation.

^a^ Subcapsular location was defined as when the index tumor abutted the liver capsule.

^b^ Subphrenic location was defined as when the index tumor was located within 1 cm from the diaphragm [6].

**Table 4:** Therapeutic outcomes after CEUS-added FI-guided RFA.

Outcome	*n* = 19
Number of ablation sessions (mean ± SD)	1.1 ± 0.2
Technical success rate (%)	94.7% (18/19)
Primary technique efficacy rate (%)	100% (19/19)
Major complication rate (%)	0% (0/19)
Local tumor progression rate (%)	10.5% (2/19)

## Data Availability

The data used to support the findings of this study are available from the corresponding author upon request.
